# Tranexamic Acid (TXA) for the Hemostatic Treatment of Post-Partum Hemorrhage (PPH): What Key Points Have We Learnt After All These Years?

**DOI:** 10.3390/jcm12196385

**Published:** 2023-10-06

**Authors:** Panagiotis Peitsidis, Christos Iavazzo, Ioannis D. Gkegkes, Antonio Simone Laganà, Sophia Makridima, Panagiotis Tsikouras

**Affiliations:** 1 Department of Obstetrics and Gynecology, Helena Venizelou Hospital, 11521 Athens, Greece; 2 Gynaecological Oncology Department, Metaxa Cancer Hospital, 18537 Piraeus, Greece; christosiavazzo@hotmail.com; 3 Athens Colorectal Laboratory, 11528 Athens, Greece; 4 Department of Colorectal Surgery, Royal Devon University Healthcare NHS Foundation Trust, Exeter EX2 5DW, UK; 5 Unit of Obstetrics and Gynecology, “Paolo Giaccone” Hospital, Department of Health Promotion, Mother and Child Care, Internal Medicine and Medical Specialties (PROMISE), University of Palermo, 90127 Palermo, Italy; 6 Department of Obstetrics and Gynecology, The Democritus University of Thrace, 68100 Alexandroupolis, Greece

Post-partum bleeding or post-partum hemorrhage (PPH) is often defined as the loss of more than 500 mL of blood after vaginal delivery or 1000 mL of blood after cesarean section following the delivery of a child [1]. Post-partum hemorrhage (PPH) is a major clinical and social-economic issue which causes 27.1% of maternal deaths globally, varying from 13.4% in high-income countries to 34% in developed countries [1]. PPH can be further classified into primary PPH (within 24 h of birth) and secondary PPH (between 24 h and 6 weeks post-partum) [2]. The essential medical management of PPH is based on the administration of uterotonics such as oxytocin, carbetocin, and methergotamine; these drugs interfere with the contraction of smooth muscle of the uterus, thus reducing blood loss. However, in recent years, an antifibrinolytic agent named tranexamic acid (TXA) has been introduced as a hemostatic agent in the treatment of PPH [3]. Tranexamic acid (ΤΧA) is a potent antifibrinolytic agent which has been included in the World Health Organization’s (WHO) list of essential medicines since 2011; the drug can be administered intravenously and per os [4]. Tranexamic acid is a synthetic analog of the amino acid lysine. It serves as an antifibrinolytic by reversibly binding to lysine receptor sites on plasminogen, thus decreasing the conversion of plasminogen to plasmin, preventing fibrin degradation and preserving the structure of the fibrin matrix [4]. Tranexamic acid was synthesized in Japan in 1962 by Okamoto et al. [5]. Tranexamic acid (TXA), also named Cyclokapron, was initially used in gynecology in several European countries, starting in 1968, as a hemostatic drug in excessive menstrual bleeding and menstrual bleeding associated with intrauterine devices (IUDs) [6,7,8]. In 1986, in Sweden, Heslop et al. [9] initially reported the use of TXA in the successful hemostatic management of PPH in a patient with a coagulopathy (Bernard Soulier syndrome); TXA was combined with other agents (Platelets-Desmopressin). Several other case reports of successful management were published later by Greer et al. [10] in 1996 in a patient with hemophilia and by As et al. [11] in 1996 in a patient with placenta accreta. The first randomized control trial (RCT) was designed in China by Yang et al. [11] in 2001 which enrolled 400 women giving birth vaginally; the patients received 1gr of TXA, and the authors reported a reduction in blood loss post-partum. However, the study had poor methodological quality due to a lack of randomization, and it was written in Chinese. The years 2009–2011 were characterized by the production of the first systematic reviews and metanalyses by Ferrer et al. [12], Novikova et al. [13], and Peitsidis et al. [14]. All metanalyses exhibited a minor decrease in PPH after 1 gr administration of TXA in trials with women undergoing vaginal delivery and cesarean section; however, all included RCTs were of low methodological quality. In 2015, McClure et al. [15] created a mathematical model to determine the impact of interventions on PPH-related maternal mortality, named the Maternal and Neonatal Directed Assessment of Technology (MANDATE) model [16]. The authors performed their model in accordance with pregnancy and mortality rates after PPH in sub-Saharan Africa. The conclusion was that with the use of only tranexamic acid in hospitals, less than 2% of the PPH mortality would be reduced. However, if tranexamic acid was available in the home and clinic settings for PPH prophylaxis and treatment, a 30% reduction (nearly 22,000 deaths per year) in PPH mortality would be possible [15].

In 2017, the largest and most important randomized, double-blind, placebo-controlled trial so far was published, the so-called WOMAN (World Maternal Antifibrinolytic) trial. The study recruited women aged 16 years and older with a clinical diagnosis of post-partum hemorrhage after a vaginal birth or caesarean section from 193 hospitals in 21 countries. The multicentric study enrolled 20,060 women who were randomly assigned to receive tranexamic acid (*n* = 10,051) or a placebo (*n* = 10,009), of whom 10,036 and 9985, respectively, were included in the analysis. The administration was 1 g (100 mg/mL) of tranexamic acid intravenously at an approximate rate of 1 mL per min. The authors reported some very important findings: death due to bleeding was significantly reduced in women given tranexamic acid (155 (1.5%) of 10,036 patients vs. 191 (1.9%) of 9985 in the placebo group, risk ratio (RR) 0.81, 95% CI 0.65–1.00; *p* = 0.045), especially in women given treatment within 3 h of giving birth (89 (1.2%) in the tranexamic acid group vs. 127 (1.7%) in the placebo group, RR 0.69, 95% CI 0.52–0.91; *p* = 0.008). These findings led to an alteration of WHO (World Health Organization) guidelines about the management of PPH, the panel emphasized that infusion of 1 g of TXA during the first 3 h reduced maternal deaths, and this effect was not beneficial after 3 h of PPH [17]. In addition, we must not forget that contraindications of TXA administration such as previous thromboembolic incidences should be taken into serious consideration.

In 2018, Ageron et al. [18] performed a metanalysis based on 40,138 patients from two randomized trials of tranexamic acid for acute severe bleeding (traumatic and post-partum hemorrhage—WOMAN and CRASH-2 trial) [19,20]. The findings exhibited that tranexamic acid significantly increased overall survival from bleeding (odds ratio (OR) 1.20, 95% CI 1.08–1.33; *p* = 0.001).

Sentilhes et al. [21] published an essential large-scale randomized control trial in 2018. The trial is known as TRAAP: TRAnexamic Acid for Preventing post-partum hemorrhage following a vaginal delivery; this was a multicentric study from 15 hospitals in France that enrolled 4079 women in labor who had a planned vaginal delivery of a single live fetus at 35 or more weeks of gestation. The women received 1 g of tranexamic acid or a placebo, administered intravenously, in addition to prophylactic oxytocin after delivery. Women in the tranexamic acid group had a lower rate of clinically significant post-partum hemorrhage than those in the placebo group (7.8% vs. 10.4%; relative risk, 0.74; 95% CI, 0.61 to 0.91; *p* = 0.004; *p* = 0.04) and also received additional uterotonic agents less often (7.2% vs. 9.7%; relative risk, 0.75; 95% CI, 0.61 to 0.92; *p* = 0.006; adjusted *p* = 0.04). The same authors published the trial TRAAP2—TRAnexamic Acid for Preventing post-partum hemorrhage after cesarean delivery [22]. In a multicenter, double-blind, randomized, controlled trial, 4551 women undergoing cesarean delivery before or during labor at 34 or more gestational weeks were allocated to groups receiving an intravenously administered prophylactic uterotonic agent and either tranexamic acid (1 g) or a placebo. The authors reported that for women who underwent cesarean section and received prophylactic uterotonic agents, tranexamic acid resulted in a significantly lower incidence of calculated estimated blood loss greater than 1000 mL, but it did not result in a lower incidence of hemorrhage-related secondary clinical outcome, and 0.4% had thromboembolic symptoms [22]. It is important to mention that TXA is a cost-effective treatment of PPH, resulting in the cost-effective analyses of the TRAAP trial [23] and TRAAP2 trial [24].

In 2023, Pacheco et al. [25] performed a large multicentric trial in US hospitals enrolling a total of 11,000 participants who underwent cesarean section delivery (5529 were allocated to the group receiving TXA, and 5471 were allocated to the placebo group). The results were not very impressive, as an estimated intraoperative blood loss of more than 1 L occurred in 7.3% of the participants in the tranexamic acid group and in 8.0% of those in the placebo group (relative risk, 0.91; 95% CI, 0.79 to 1.05), and the main conclusion was that prophylactic use of tranexamic acid during cesarean delivery did not lead to a significantly lower risk of maternal death or blood transfusion than the placebo [25].

Tranexamic acid is an old agent that remains an essential drug in the hemostatic management of post-partum hemorrhage, and it is without doubt cost-effective. The earlier the administration is provided, the more maternal mortality and blood loss are decreased, though we must consider combined therapies and multidisciplinary approaches. It is recommended that numerous systematic reviews, including updated network metanalyses, should be published annually regarding tranexamic acid and post-partum hemorrhage.

We are awaiting the results of the multicentric WOMAN-2 trial, which is currently running, and it is strongly believed that WOMAN-2 will provide reliable evidence about the effects of TXA in women with anemia [26].
